# Supplementary material for: The chromatin organization of a chlorarachniophyte nucleomorph genome
Source: Genome Biol. 2022 Mar 1;23:65. doi: 10.1186/s13059-022-02639-5 (PMC8887012; doi:10.1186/s13059-022-02639-5)
Supplement: Supplementary file 1 — Additional file 1: Fig. S1. Relative genomic copy numbers of the different B. natans genomic compartments. Shown are mappability-corrected RPKM values for an input sample for each chromosome/contig. Fig. S2. Relationship between transcript levels (based on RNA-seq) and KAS-seq in the nucleomorph. Shown are RNA-seq TPM values (using RNA-seq data from GEO accession GSE115762) and KAS-seq RPM values. Several highly expressed genes such as Hsp70, Hsp90, DnaK and ribosomal RNAs are highlighted. Fig. S3. Relationship between chromatin accessibility and active transcription as measured by KAS-seq in the B. natans nuclear genome. (A) Correlation between ATAC-seq signal over promoters and KAS-seq signal over promoters. (B) Correlation between ATAC-seq signal over promoters and KAS-seq signal over gene bodies. Fig. S4. Impact of different Hi-C normalization methods on the quantification of Hi-C trans contacts between different compartments. (A) KR normalization (B) No normalization (C) Coverage normalization (VC) (D) Coverage normalization (VC SQRT). [file 13059_2022_2639_MOESM1_ESM.pdf]

Supplementary Materials

Supplementary Figures

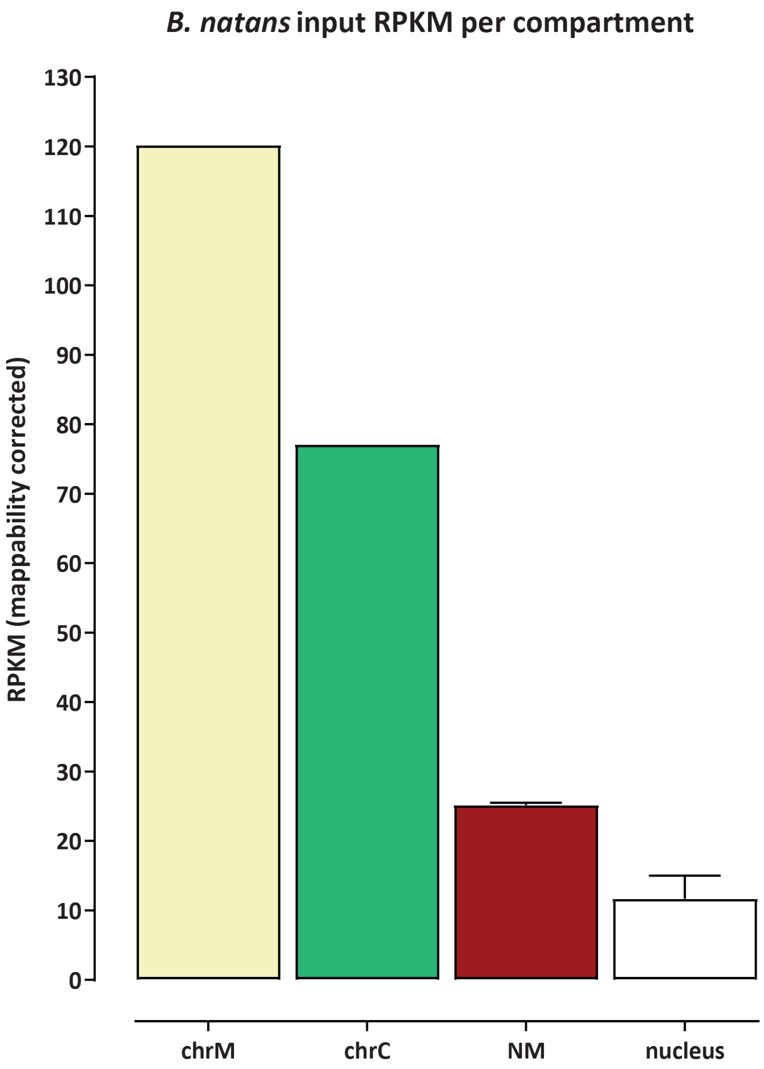

Figure S 1: Relative genomic copy numbers of the different *B. natans* genomic compartments. Shown are mappability-corrected RPKM values for an input sample for each chromosome/contig.

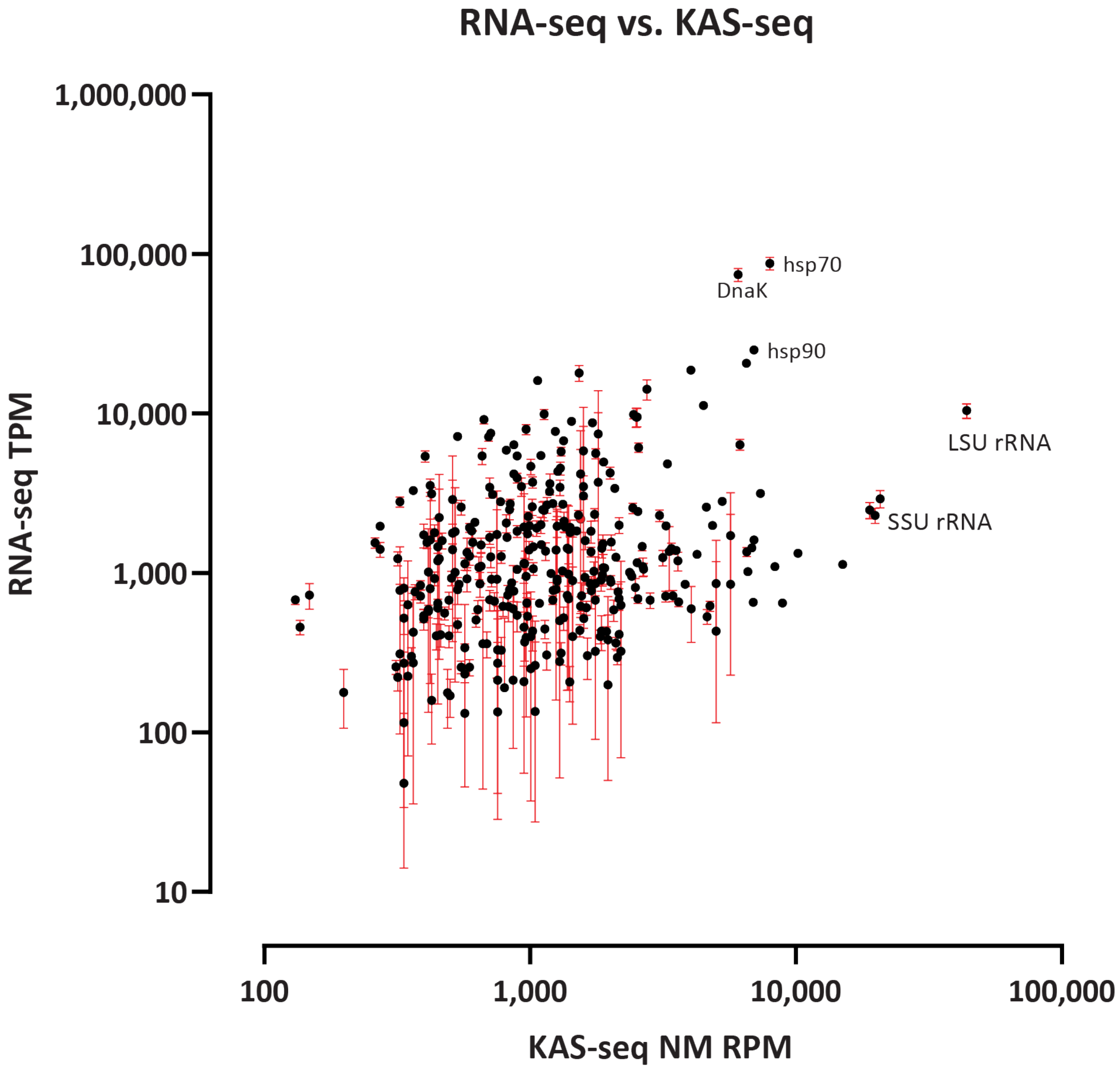

Figure S 2: Relationship between transcript levels (based on RNA-seq) and KAS-seq in the nucleomorph. Shown are RNA-seq TPM values (using RNA-seq data from GEO accession GSE115762) and KAS-seq RPM values. Several highly expressed genes such as *Hsp70*, *Hsp90*, *DnaK* and ribosomal RNAs are highlighted

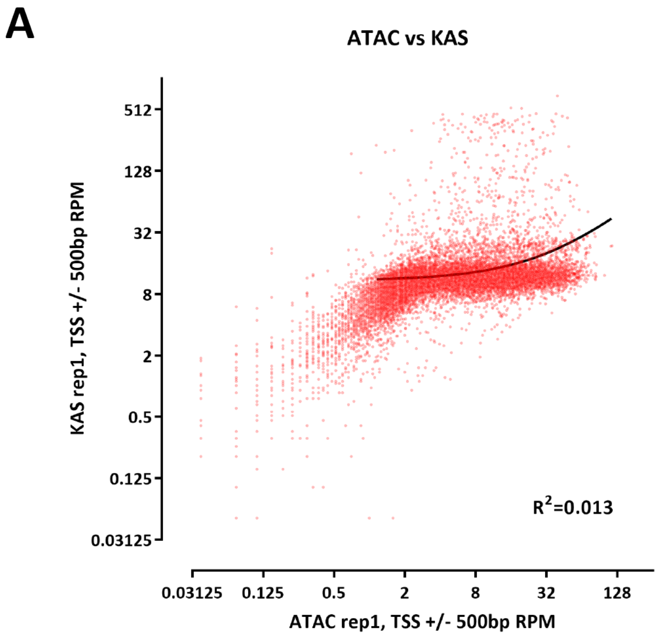

Figure S 3: Relationship between chromatin accessibility and active transcription as measured by KAS-seq in the *B. natans* nuclear genome. (A) Correlation between ATAC-seq signal over promoters and KAS-seq signal over promoters. (B) Correlation between ATAC-seq signal over promoters and KAS-seq signal over gene bodies.

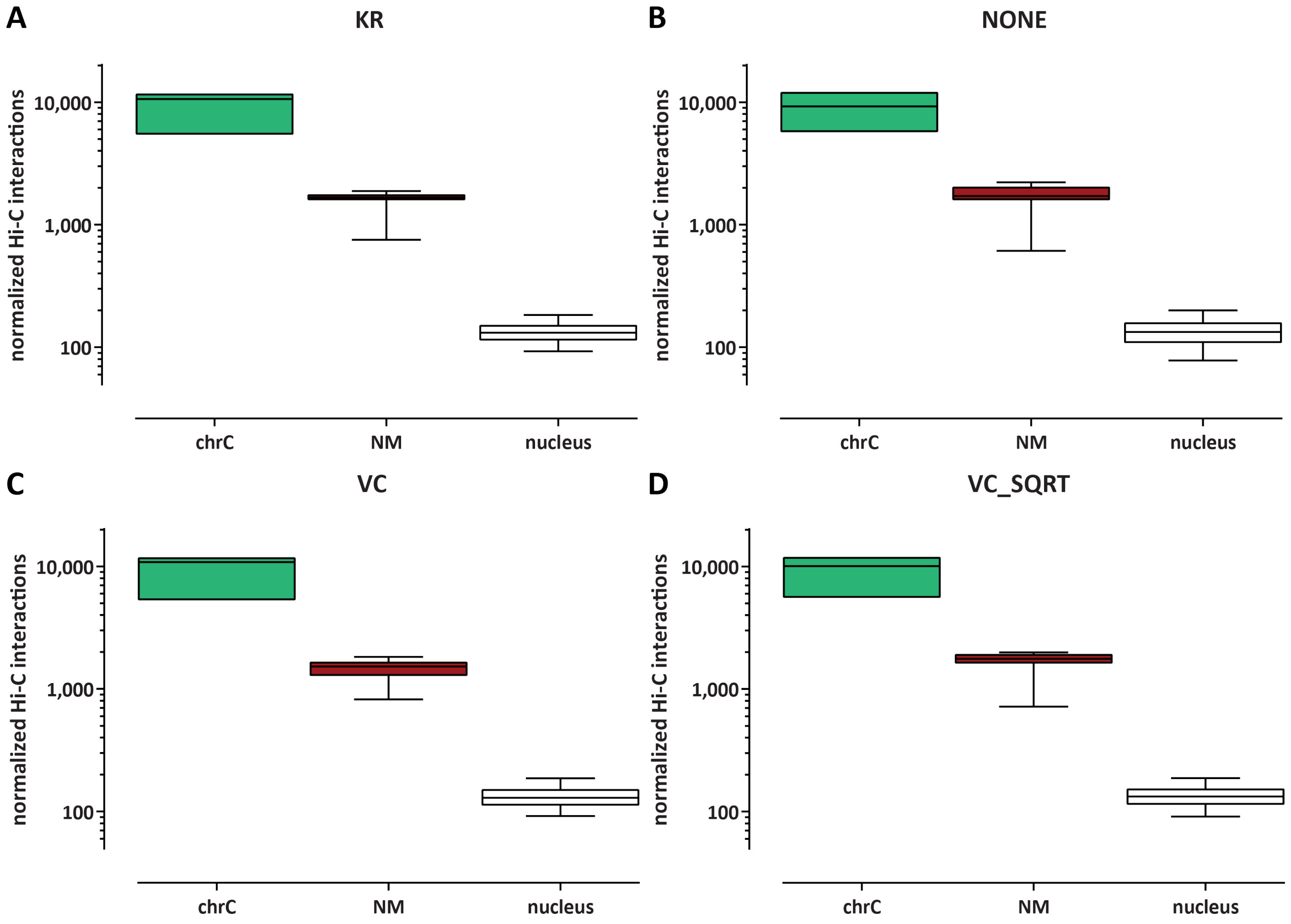

Figure S 4: Impact of different Hi-C normalization methods on the quantification of Hi-C *trans* contacts between different compartments. (A) KR normalization (B) No normalization (C) Coverage normalization (VC) (D) Coverage normalization (VC_SQRT)
