# Supplementary material for: The chromatin organization of a chlorarachniophyte nucleomorph genome
Source: Genome Biol. 2022 Mar 1;23:65. doi: 10.1186/s13059-022-02639-5 (PMC8887012; doi:10.1186/s13059-022-02639-5)
Supplement: Supplementary file 2 — Additional file 2. Review history. [file 13059_2022_2639_MOESM2_ESM.docx]

Review History

**First round of review**

**Reviewer 1**

**Are you able to assess all statistics in the manuscript, including the appropriateness of statistical tests used?**

Yes, and I have assessed the statistics in my report.

**Comments to author:**

Chlorarachniophytes have complex plastids acquired by secondary endosymbiosis of green algae, and interestingly those plastids possess a relict nucleus, so-called nucleomorph, derived from the endosymbiont. Nucleomorphs contain a highly reduced genome to compare with free living green algal ones. Nucleomorph genomes consist of three short chromosomes that exhibit an extremely gene-dense structure with shrinking noncoding regions (very short intergenic spacers and ultrasmall spliceosomal introns). Several recent studies reported that nucleomorph genes are more abundantly transcribed than nuclear genes based on RNA-seq data. However, regulation mechanism of nucleomorph gene expression remain unclear. In this study, Marinov and collegues performed three sequence analyses (ATAC-seq, KAS-seq, and Hi-C-seq) to understand the reason for the active expression of nucleomorph genes.

My main suspicion is about the result of Hi-C experiments. The Hi-C map (3D genomic organization) indicates that the mitochondrial DNA contacts with the plastid and nucleomorph DNA, even though those DNA are physically separated by multiple membranes. The authors inferred that the crosslinks of organelle genomes between different compartments would be caused by fixation artifacts. If this is true, the other interactions, such as nucleomorph telomeric regions, may also be experimental artifacts. To deny this possibility, I think that the authors need to provide additional data to support the result of Hi-C experiments (e.g., high-resolution imaging approaches as the authors suggested, page 8 right line 40).

Page 6, left line 56. "These observations, based on measuring actual active transcription, corroborate previous reports, based on transcriptomic analysis, of high, pervasive, and largely uniform transcriptional activity over most of the nucleomorph genome." In my knowledge, most genes are actively transcribed across nucleomorph genomes, but not uniformly. Hirakawa et al., (2014) MBE, 31:1437-43 reported that several nucleomorph genes, such as hsp70 & hsp90, were extremely highly expressed in Bigelowiella natans, and there was a hundred times difference in expression level between top and bottom. Therefore, I'm curious about the relationship between expression level and KAS-seq signal for each nucleomorph gene, because several peaks of KAS-seq profile can be observed in Fig 3C.

Page 7, left line 54. "the telomeric regions of all nucleomorph chromosomes physically associate with each other, forming a telomeric network of interactions". Is it possible to detect an interaction between the same sequences in different regions by Hi-C seq? For example, if a telomeric sequence (TAGACCC)n of chromosome#1 is interacted with a telomeric sequence (TAGACCC)n of chromosome#2, how can you identify this interaction from the Hi-C sequence data?

Page 7, left line 59. "enriched interchromosomal interactions in nucleomorphs appear to be only telomeric". I'm wondering how the authors distinguished between the 'interchromomsomal' and 'intrachromosomal' interactions, because the three chromosomes have the completely identical telomeric sequences.

Page 8, left line 16. "While both the plastid and the mitochondrial genomes exist in high copy numbers, the nucleomorph genome has the same copy number as the nuclear genome." A previous study (Hirakawa & Ishida 2014. GBE 6:974-980) measured the genome copy numbers in B. natans by quantitative PCR; the nuclear genome is haploid, the nucleomorph genome is diploid, and plastid and mitochondrial genomes carry approximately 18-40 and 30-50 copies, respectively. Please show the estimated copy number for each genome based on read coverage, and compare to the previous study.

**Reviewer 2**

**Are you able to assess all statistics in the manuscript, including the appropriateness of statistical tests used?**

No, I do not feel adequately qualified to assess the statistics.

**Comments to author:**

This is a very interesting and important study that I would have liked to do myself. Peer-reviewing it is the closest thing to that, so I'm thankful for this.

Nucleomorph are among the most fascinating eukaryotic genomes. They illustrate in exquisite detail the effects of extreme compaction, and the constraints that genome architecture imposes against it, up until the evolutionary singularity that constitutes the disappearance of the nucleomorph. But in a more fundamental side, the discovery of the nucleomorphs provided the most eloquent and indisputable smoking gun demonstrating the origin of secondary plastids. This study is the most important in the field after the initial wave of nucleomorph whole genome sequences published over a decade ago.

The introduction is well written and does a good job summarizing the key aspects relevant to nucleomorphs, justifying why the work presented here is needed. The authors may want to consider some references that mentioned and/or discussed the issues of the lacking CTD (Stump and Ostrozhynska 2013, Transcription; Lane et al. 2007 PNAS) and transcriptional regulation (Williams et al. 2005 PNAS).

While a priori one would be very careful and perhaps skeptical when analyzing the ATAC-seq data and other assays performed on the highly unusual nucleomorph genome, the authors take advantage of the nuclear genome as a 'built-in' control that allows ruling out potential artifacts. I wonder if the authors considered the effect of copy number (i.e., the number of genomes per cell) of mt vs pl vs nm vs nu genomes on ATAC-seq enrichment. It shouldn't for nu vs nm but maybe for mt and plastid. More generally, are there potential issues with unequal access to the compartments due to differential lysis?

Figures 3A and 3C are maps at different horizontal scales; what about vertical? Hard to get an idea of how the signal between compartments compare to each other.

I'm intrigued by the nucleosome depletion upstream of promoters and what could be the driver of this pattern. I wonder if a sequence-independent feature such as periodicity or higher-order folding may cause this.

Overall the study opens a can bursting with worms in terms of questions and follow up avenues, which is very exciting. I strongly commend the authors for providing very detailed protocols and making the custom scripts available. Considering that this study makes use of state of the art technology but on a novel system (the nm), this is very important.

Finally, I caught a typo in line 33, page 10 (Purification)

**Authors Response**

**Point-by-point responses to the reviewers’ comments:**

Reviewer #1:

Chlorarachniophytes have complex plastids acquired by secondary endosymbiosis of green algae, and interestingly those plastids possess a relict nucleus, so-called nucleomorph, derived from the endosymbiont. Nucleomorphs contain a highly reduced genome to compare with free living green algal ones. Nucleomorph genomes consist of three short chromosomes that exhibit an extremely gene-dense structure with shrinking noncoding regions (very short intergenic spacers and ultrasmall spliceosomal introns). Several recent studies reported that nucleomorph genes are more abundantly transcribed than nuclear genes based on RNA-seq data. However, regulation mechanism of nucleomorph gene expression remain unclear. In this study, Marinov and collegues performed three sequence analyses (ATAC-seq, KAS-seq, and Hi-C-seq) to understand the reason for the active expression of nucleomorph genes.

*Response: We thank the reviewer for the thoughtful comments and suggestions.*

My main suspicion is about the result of Hi-C experiments. The Hi-C map (3D genomic organization) indicates that the mitochondrial DNA contacts with the plastid and nucleomorph DNA, even though those DNA are physically separated by multiple membranes. The authors inferred that the crosslinks of organelle genomes between different compartments would be caused by fixation artifacts. If this is true, the other interactions, such as nucleomorph telomeric regions, may also be experimental artifacts. To deny this possibility, I think that the authors need to provide additional data to support the result of Hi-C experiments (e.g., high-resolution imaging approaches as the authors suggested, page 8 right line 40).

*Response: We understand the reviewer’s concern, but we believe there might be some confusion regarding our interpretation of the nature of the observed intercompartmental Hi-C contacts. We believe these likely arise as a result of the physical proximity of the compartments to each other. This likely leads to some level of crosslinking between chromosomes belonging to each, which is substantially lower than that observed within the compartment but also considerably higher than what would be observed if the compartments are not in such physical proximity. Therefore we don’t believe these interactions to be “artifactual” in the sense that they are an experimental artifact that does not reflect actual physical proximity, but we do not claim nor do we believe that the genomes from different compartments make direct physical contact in the living cell. Our aim in the text was to simply comment on this observation that there is an elevated frequency of observed contacts between certain compartments in the Hi-C maps, and that this observation is unexpected based on the topological organization of the secondary symbiosis that gave rise to chlorarachniophytes (i.e. the mitochondria and the host nucleus are separated by fewer membranes than mitochondria and the nucleomorph) . Existing electron microscopy imaging data (Response to Reviewers Figures 11 and 22 ) supports that observation, as mitochondria are observed close to the nucleomorph in those images.*

Page 6, left line 56. "These observations, based on measuring actual active transcription, corroborate previous reports, based on transcriptomic analysis, of high, pervasive, and largely uniform transcriptional activity over most of the nucleomorph genome." In my knowledge, most genes are actively transcribed across nucleomorph genomes, but not uniformly. Hirakawa et al., (2014) MBE, 31:1437-43 reported that several nucleomorph genes, such as hsp70 & hsp90, were extremely highly expressed in Bigelowiella natans, and there was a hundred times difference in expression level between top and bottom. Therefore, I'm curious about the relationship between expression level and KAS-seq signal for each nucleomorph gene, because several peaks of KAS-seq profile can be observed in Fig 3C.

*Response: We thank the reviewer very much for pointing this out. We have changed the manuscript accordingly and added a new Supplementary Figure showing the relationship between KAS-seq and RNA-seq (using publicly available RNA-seq datasets). It is also shown here as Response To Reviewers Figure 3).*

Page 7, left line 54. "the telomeric regions of all nucleomorph chromosomes physically associate with each other, forming a telomeric network of interactions". Is it possible to detect an interaction between the same sequences in different regions by Hi-C seq? For example, if a telomeric sequence (TAGACCC)n of chromosome#1 is interacted with a telomeric sequence (TAGACCC)n of chromosome#2, how can you identify this interaction from the Hi-C sequence data?

Page 7, left line 59. "enriched interchromosomal interactions in nucleomorphs appear to be only telomeric". I'm wondering how the authors distinguished between the 'interchromomsomal' and 'intrachromosomal' interactions, because the three chromosomes have the completely identical telomeric sequences.

*Response: Hi-C analysis is carried out by excluding multimapping reads and over relatively large bins of genomic sequences (5-kb or 1-kb in our case). Interactions are aggregated over all reads mapping to these bins. The nucleomorph telomeric regions themselves are not uniquely mappable, and this is reflected in the “white” margins of the squares for each nucleomorph chromosome. However, the subtelomeric regions are uniquely mappable and the subtelomereto-subtelomere interactions are clearly visible there – Hi-C contacts are not tightly localized but form a continuum of enrichment around the actual interaction area (if that interaction area is in fact highly localized) or it is in fact the case that the interactions are not highly localized and rather broad in their span. We also note that such telomereto-telomere interactions (as inferred by subtelomere-to-subtelomere enrichment) are observed in Hi-C datasets for many other species and are a common feature of Hi-C maps. Therefore we believe that this evidence from the subtelomeric regions is telling us beyond reasonable doubt that the telomeres are also interacting with each other. We have added a clarification in the text regarding subtelomeres.*

Page 8, left line 16. "While both the plastid and the mitochondrial genomes exist in high copy numbers, the nucleomorph genome has the same copy number as the nuclear genome." A previous study (Hirakawa & Ishida 2014. GBE 6:974-980) measured the genome copy numbers in B. natans by quantitative PCR; the nuclear genome is haploid, the nucleomorph genome is diploid, and plastid and mitochondrial genomes carry approximately 18-40 and 30-50 copies, respectively. Please show the estimated copy number for each genome based on read coverage, and compare to the previous study.

*Response: We thank the reviewer for pointing this out and have revised the manuscript accordingly. We had calculated the number of reads mapping to chromosomes in Hi-C samples (processed as an input) as an intermediate step in the enrichment calculations in Figure 1. We have now updated the manuscript with a figure showing this explicitly (also shown here as Response To Reviewers Figure 4).*

Reviewer #2:

This is a very interesting and important study that I would have liked to do myself. Peer-reviewing it is the closest thing to that, so I'm thankful for this.

Nucleomorph are among the most fascinating eukaryotic genomes. They illustrate in exquisite detail the effects of extreme compaction, and the constraints that genome architecture imposes against it, up until the evolutionary singularity that constitutes the disappearance of the nucleomorph. But in a more fundamental side, the discovery of the nucleomorphs provided the most eloquent and indisputable smoking gun demonstrating the origin of secondary plastids. This study is the most important in the field after the initial wave of nucleomorph whole genome sequences published over a decade ago.

*Response: We thank the reviewer for the thoughtful suggestions and comments and are glad that the manuscript has been of interest.*

The introduction is well written and does a good job summarizing the key aspects relevant to nucleomorphs, justifying why the work presented here is needed. The authors may want to consider some references that mentioned and/or discussed the issues of the lacking CTD (Stump and Ostrozhynska 2013, Transcription; Lane et al. 2007 PNAS) and transcriptional regulation (Williams et al. 2005 PNAS).

*Response: We thank the reviewer for these useful suggestions (we had indeed missed some of those references). We have updated the manuscript accordingly.*

While a priori one would be very careful and perhaps skeptical when analyzing the ATAC-seq data and other assays performed on the highly unusual nucleomorph genome, the authors take advantage of the nuclear genome as a 'built-in' control that allows ruling out potential artifacts. I wonder if the authors considered the effect of copy number (i.e., the number of genomes per cell) of mt vs pl vs nm vs nu genomes on ATAC-seq enrichment. It shouldn't for nu vs nm but maybe for mt and plastid. More generally, are there potential issues with unequal access to the compartments due to differential lysis?

*Response: The reviewer raises several important points. One of them (the relative ploidy) is also raised by Reviewer #1 and we have answered it (and revised the manuscript with the addition of the necessary analysis) in response to that previous question. Differential lysis is something we have considered as a possibility. At present we do not have a good way to control for it, but it is likely not a major concern for the lysis of Hi-C samples as from those we recover signal proportional to the expected relative ploidy of the genomic and nucleomorph compartments (see Response To Reviewers Figure 4). It is possible that differential lysis might bias results in the ATAC-seq datasets. However, if this was indeed happening we might expect such a bias to decrease the relative ATAC signal obtained from the nucleomorph. Such a perturbation would not change the main conclusions of our study regarding the high overall accessibility of the nucleomorph genome. However, based on electron microscopy images (also see our response to Reviewer #1), the nucleomorph plus plastid compartment is actually of comparable or even larger size than the nucleus, thus it is unlikely that it is preferentially depleted during the ATAC protocol.*

Figures 3A and 3C are maps at different horizontal scales; what about vertical? Hard to get an idea of how the signal between compartments compare to each other.

*Response: We have added y-axis labels to all browser snapshots.*

I'm intrigued by the nucleosome depletion upstream of promoters and what could be the driver of this pattern. I wonder if a sequence-independent feature such as periodicity or higher-order folding may cause this.

*Response: We share the reviewer’s interest in the mechanisms of generating the open promoter structures. Unfortunately what makes nucleomorphs unique – their extreme miniaturization – also makes it difficult to do sequence analysis as there are just too few examples to learn potentially weak sequence pattern preferences. We expect the application of analogous methods in more chlorarachniophyte species to help with the answers to these questions, as accumulating more such datasets will allow us to look for conserved patterns across sufficiently many examples.*

*Overall the study opens a can bursting with worms in terms of questions and follow up avenues, which is very exciting. I strongly commend the authors for providing very detailed protocols and making the custom scripts available. Considering that this study makes use of state of the art technology but on a novel system (the nm), this is very important.*

Finally, I caught a typo in line 33, page 10 (Purification).

*Response: We thank the reviewer for spotting this typo. It has been corrected.*

**Second round of review**

**Reviewer 1**

The authors have addressed all my concerns adequately, and additional data further support the validity of this interesting study.

Let me point out one thing: I’d like to suggest the authors to remove the sentence “We note that existing electron microscopy images of the ultrastructural organization of the B. natans cells also suggest frequent greater physical proximity between the nucleomorph and mitochondria than between the nucleus and mitochondria” in page 8. The authors provided two TEM pictures of B. natans in the response letter. The picture of Hopkins seems to indicate the physical proximity between a mitochondrion and nucleomorphs. In contrast, the Moestrup’s picture shows that a mitochondrion (m) is located near the host nucleus (N) rather than the nucleomorph (n). Therefore, I think that the position of mitochondria is unstable in the B. natans cell, and the physical proximity of mitochondria and nucleomorph is ambiguous.

In my understanding, elevated Hi-C contacts were observed among mitochondrial, plastid, and nucleomorph genomes. These three genomes exhibit multiple copy numbers and show higher accessibility (based on ATAC-seq) compared with the nuclear genome. Is there a possibility that Hi-C contacts among the three genomes are related to their high accessibility/chromatin state/high copy number?

Typo: please change “nucleomoprh” to “nucleomorph” in page 1 (Abstract), page 8 and page 10.

**Reviewer 2**

The authors have satisfied all my concerns. Congrats on this nice paper.

**Authors Response**

**Point-by-point responses to the reviewers’ comments:**

Reviewer 2#:

The authors have addressed all my concerns adequately, and additional data further support the validity of this interesting study.

*Response: We thank the reviewer for the suggestions and comments.*

Let me point out one thing: I’d like to suggest the authors to remove the sentence “We note that existing electron microscopy images of the ultrastructural organization of the B. natans cells also suggest frequent greater physical proximity between the nucleomorph and mitochondria than between the nucleus and mitochondria” in page 8. The authors provided two TEM pictures of B. natans in the response letter. The picture of Hopkins seems to indicate the physical proximity between a mitochondrion and nucleomorphs. In contrast, the Moestrup’s picture shows that a mitochondrion (m) is located near the host nucleus (N) rather than the nucleomorph (n). Therefore, I think that the position of mitochondria is unstable in the B. natans cell, and the physical proximity of mitochondria and nucleomorph is ambiguous.

*Response: We have revised the text accordingly.*

In my understanding, elevated Hi-C contacts were observed among mitochondrial, plastid, and nucleomorph genomes. These three genomes exhibit multiple copy numbers and show higher accessibility (based on ATAC-seq) compared with the nuclear genome. Is there a possibility that Hi-C contacts among the three genomes are related to their high accessibility/chromatin state/high copy number?

*Response: We had this concern ourselves and thus examined all possible normalization strategies that account for copy number and accessibility, obtaining identical results with all of them (Supplementary Figure 4). Thus it is unlikely that such biases are behind the observed increased contact frequency.*

Typo: please change “nucleomoprh” to “nucleomorph” in page 1 (Abstract), page 8 and page 10.

*Response: We thank the reviewer for spotting this typo, and have corrected it.*
